# Spatial and Temporal Changes in Prevalence of Obesity Among Chinese Children and Adolescents, 1985–2005

**DOI:** 10.5888/pcd16.190290

**Published:** 2019-12-12

**Authors:** Peng Jia, Shuang Ma, Xin Qi, Youfa Wang

**Affiliations:** 1 GeoHealth Initiative, Department of Earth Observation Science, Faculty of Geo-Information Science and Earth Observation (ITC), University of Twente, Enschede, Netherlands; 2 International Initiative on Spatial Lifecourse Epidemiology (ISLE), Enschede, Netherlands; 3 Global Health Institute, School of Public Health, Xi’an Jiaotong University Health Science Center, Xi’an, Shaanxi, China; 4 Fisher Institute of Health and Well-Being, Department of Nutrition and Health Sciences, College of Health, Ball State University, Muncie, Indiana

**Figure:**
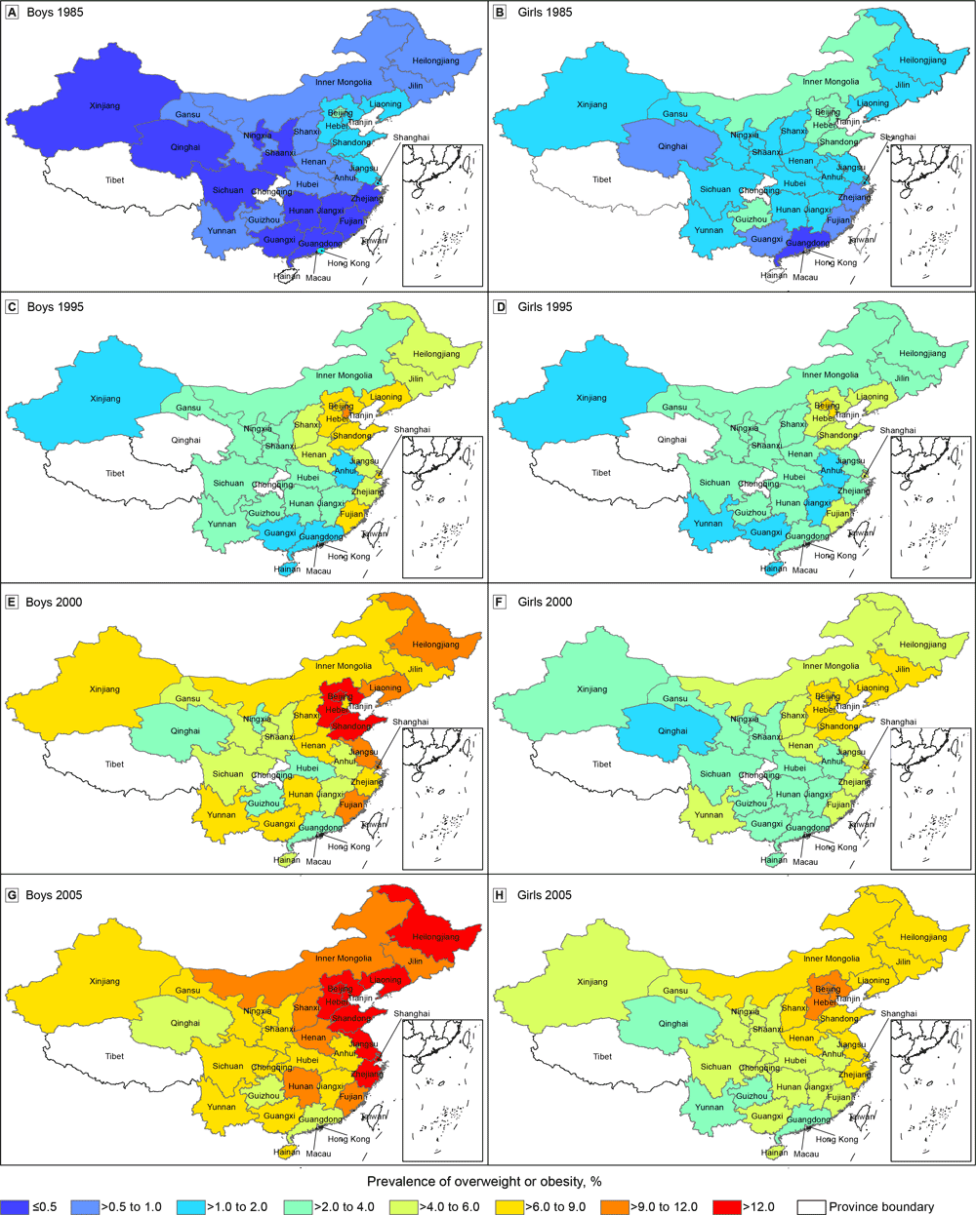
Differences in the prevalence of overweight/obesity among children and adolescents in the 30 mainland provinces of China in 1985, 1995, 2000, and 2005. Data were stratified by sex and year. Data were derived from 4 waves of the Chinese National Survey on Student’s Constitution and Health (5). Areas with no color were not included in the survey. Overweight/obesity was defined as body mass index ≥85th percentile (6).

**Table:** 

Province	1985		1995		2000		2005	
	Boys	Girls	Boys	Girls	Boys	Girls	Boys	Girls
Anhui	0.59	1.32	1.67	2.00	5.31	4.00	6.42	4.62
Beijing	2.92	2.99	8.18	6.96	13.0	8.61	16.41	10.09
Chongqing	Not available	Not available	Not available	Not available	Not available	Not available	7.12	4.63
Fujian	0.33	0.65	6.28	4.14	10.39	5.06	10.61	5.40
Inner Mongolia	0.60	2.08	3.47	2.85	6.78	4.88	9.97	7.06
Gansu	0.53	1.32	2.44	2.66	5.32	3.8	7.71	4.97
Guangdong	0.26	0.48	1.81	2.11	3.86	3.31	5.58	3.15
Guangxi	0.22	0.67	1.71	1.32	6.14	3.58	6.39	4.46
Guizhou	0.60	2.06	2.36	3.94	2.92	2.53	4.41	3.87
Hainan	Not available	Not available	1.76	1.32	5.41	2.95	7.05	4.25
Hebei	1.17	2.13	7.36	5.03	12.50	8.32	12.45	9.03
Heilongjiang	0.74	1.63	5.28	3.92	9.06	5.78	14.74	8.40
Henan	0.64	1.28	4.17	3.43	8.02	5.62	11.25	7.72
Hubei	0.63	1.41	3.72	2.03	3.81	2.53	7.54	5.14
Hunan	0.40	1.10	3.29	2.94	6.28	3.31	9.05	4.12
Jiangsu	1.10	1.88	4.45	3.52	10.18	5.67	13.95	8.51
Jiangxi	0.42	1.16	2.48	1.98	5.28	3.01	7.74	4.88
Jilin	0.77	1.18	4.57	3.39	6.79	6.57	9.86	7.27
Liaoning	1.12	1.66	6.67	5.03	9.68	6.40	12.89	8.72
Ningxia	0.31	1.42	2.47	2.28	3.14	3.72	7.93	5.80
Qinghai	0.23	0.78	Not available	Not available	2.83	1.81	4.43	2.74
Shaanxi	0.35	1.01	3.23	2.84	5.69	3.83	7.22	4.28
Shandong	1.91	2.10	7.53	4.89	12.99	7.87	14.29	8.86
Shanghai	1.66	1.54	8.70	5.48	11.3	6.47	14.63	8.09
Shanxi	0.83	1.67	4.45	3.65	8.28	5.25	9.9	6.59
Sichuan	0.35	1.58	2.27	2.64	4.47	2.98	6.64	4.25
Tianjin	1.69	2.21	10.43	7.36	7.41	7.91	15.68	10.03
Xinjiang	0.48	1.05	1.94	1.83	7.40	2.92	8.89	4.52
Yunnan	0.59	1.50	2.79	1.83	6.16	4.57	6.66	3.63
Zhejiang	0.33	0.78	4.96	3.11	8.56	5.39	12.04	6.79

## Background

Obesity poses a threat to public health worldwide, and its prevalence has increased rapidly in some developing countries (1). Childhood obesity is especially deserving of the attention of the public health community, because it impairs the cognitive, behavioral, and social-emotional development of children and tends to persist into adolescence and adulthood (2,3). Identifying geographical disparities and clusters of prevalence is important for understanding the causes of childhood obesity and for designing and targeting interventions in areas of most need. The prevalence of childhood obesity has increased remarkably in China in the past 3 decades. This increase may be due to policy, socioeconomic, environmental, and lifestyle changes that occurred from 1985 through 2005 (4). We used available data from 4 waves of the Chinese National Survey on Student’s Constitution and Health (CNSSCH) to examine spatiotemporal variations in the prevalence of childhood obesity during that period.

## Data Sources and Map Logistics

Data on the combined prevalence of overweight/obesity among children in each province of China were derived from 4 waves of CNSSCH, in 1985, 1995, 2000, and 2005 (5). The CNSSCH survey was jointly conducted by the Ministry of Education, the Ministry of Health, the Ministry of Science and Technology, the State of Nation Affairs, and the State Sports General Administration in China. To date, it is the largest representative health survey of school-aged children and adolescents in China. The sample includes Han children and adolescents aged 7 to 18 in 30 mainland provinces, except Tibet, where Han Chinese do not constitute an ethnic majority. Data for Qinghai Province in 1995 were missing. Hainan Province and Chongqing City became independent administrative units of Guangdong Province (in 1988) and Sichuan Province (in 1997), respectively, so they shared the same prevalence with the units they belonged to before independence.

Overweight and obesity were defined according to age–sex-specific body mass index (BMI) cutpoints. We used standards set by the China Obesity Working Group: BMI from the 85th percentile to less than the 95th percentile was used to define overweight and BMI in the 95th percentile or greater was used to define obesity (6). We categorized prevalence of overweight/obesity into 8 categories (≤0.5%, >0.5% to 1.0%, >1.0% to 2.0%, >2.0% to 4.0%, >4.0% to 6.0%, >6.0% to 9.0%, >9.0% to 12.0% and >12.0%).

We stratified the prevalence of overweight/obesity by sex and province and mapped it for each wave by using ArcGIS version 10.6.1 (Esri). We analyzed the temporal trend of overweight/obesity prevalence in each province separately for boys and girls, and we examined the significance of temporal changes by using the χ^2^ test in SPSS version 18.0 (IBM Corp). We examined the spatiotemporal clustering patterns of province-level prevalence for the 4 waves of data in SaTScan version 9.6 (Martin Kulldorff and Information Management Services Inc), and we used the Bernoulli model for the probability model. We set up the program to search for areas of an exceptionally high prevalence of overweight/obesity, with a radius of up to 500 km. To ensure that the 500-km radius was not restricted, a cluster was allowed to contain up to 50% of the population at risk of overweight/obesity in China. A limitation of the map was that it was subject to changes in spatial scale, usually described as the modifiable areal unit problem (7).

## Highlights

We found large regional differences in the prevalence of overweight/obesity among children and adolescents in China, and we found changes in patterns over time. In 1985, the prevalence of overweight/obesity among boys was highest in Beijing (2.9%), followed by Shandong (1.9%), Tianjin (1.7%), and Shanghai (1.7%), and lowest in Guangxi (0.2%), followed by Qinghai (0.2%) and Guangdong (0.3%). We found a similar pattern among girls in the same year: the prevalence was highest in Beijing (3.0%), followed by Tianjin (2.2%), Hebei (2.1%), and Shandong (2.1%), and lowest in Guangdong (0.5%), followed by Fujian (0.6%) and Guangxi (0.7%). In 1995, 3 municipalities (Beijing, Tianjin, and Shanghai) were also province-equivalent units with the highest prevalence of overweight/obesity among both boys (Tianjin, 10.4%; Shanghai, 8.7%; Beijing, 8.2%) and girls (Tianjin, 7.4%; Beijing, 7.0%; Shanghai, 5.5%). The prevalence of overweight/obesity was still high in Shandong (boys, 7.5%; girls, 4.9%) and Hebei (boys, 7.4%; girls, 5.0%). Guangxi (boys, 1.7%; girls, 1.3%) and Guangdong (boys, 1.8%; girls, 2.1%) remained among provinces with the lowest prevalence of overweight/obesity. Hainan, joining the survey in 1995, had a very low prevalence of overweight/obesity among both boys (1.8%) and girls (1.3%).

In 2000, the prevalence of overweight/obesity was highest among both sexes in Beijing (boys, 13.0%; girls, 8.6%). In Shanghai, the prevalence was higher among boys (11.3%) than girls (6.5%), and in Tianjin, the prevalence was higher among girls (7.9%) than boys (7.4%). Shandong (boys, 13.0%; girls, 7.9%) and Hebei (boys, 12.5%; girls, 8.3%) remained among the provinces with the highest prevalence. Qinghai (boys, 2.8%; girls, 1.8%) and Guizhou (boys, 2.9%; girls, 2.5%) had the lowest prevalence among both sexes. In 2005, Tianjin was back among provinces with the highest prevalence among both sexes (boys, 15.7%; girls, 10.0%), second only to Beijing (boys, 16.4%; girls, 10.1%). By 2005, the prevalence in Heilongjiang (boys, 14.7%; girls, 8.4%) had reached levels similar to those in Beijing and Tianjin, and the prevalence remained high in Shanghai (boys, 14.6%; girls, 8.1%). On the other end of the spectrum in 2005, Guizhou, Qinghai, and Guangdong were consistently among the provinces with the lowest prevalence among both boys (Guangdong, 5.6%; Qinghai, 4.4%; Guizhou, 4.4%) and girls (Guizhou, 3.9%; Guangdong, 3.2%; Qinghai, 2.7%).

The prevalence of overweight/obesity among both boys and girls in all provinces increased during 1985–2005 (*P* < .001), and the gap between the sexes widened. The prevalence was higher among boys than girls in all provinces, except Shanghai, during the study period. The center of clustering regions of overweight/obesity gradually shifted toward the southeast (moving from Beijing to Shandong).

## Action

This national map demonstrated, for the first time, the nationwide spatiotemporal changes in childhood obesity at the province level in China. It showed a clear growth trend in all provinces during 1985–2005, with the gap between boys and girls gradually expanding. Factors such as socioeconomic status, behavioral patterns, and social culture may have played a role in the rise of obesity. With the development of China’s economy and changes in policies, the variety and quantity of food have been greatly improved, the fast food industry has grown rapidly, and children and adolescents have had increasing amounts of pocket money (8,9). These factors make it easier for children and adolescents to consume excess food and beverages. Also, many policy, socioeconomic, environment, and lifestyle changes took place during the study period: for example, an increase in purchasing power and availability of goods and services, the abolition of goods-rationing systems, the importation of Western fast food, and growth in car ownership. Future public health approaches in China should use environmental and policy strategies to fight the expanding epidemic of childhood obesity, such as improving built environments that encourage physical activity, establishing regulations on fast food expansion and marketing, improving nutrition education, and changing social norms about healthy body image and weight through social marketing and media (10).
